# Hospitalisation for lower respiratory tract infection is associated with an increased incidence of acute myocardial infarction and stroke in tropical Northern Australia

**DOI:** 10.1038/s41598-021-86301-3

**Published:** 2021-03-25

**Authors:** A. Pak, D. P. Eisen, E. S. McBryde, O. A. Adegboye

**Affiliations:** 1 Australian Institute of Tropical Health and Medicine, James Cook University, 1 James Cook Drive, Douglas, QLD 4814 Australia; 2 College of Medicine and Dentistry, James Cook University, 1 James Cook Drive, Douglas, QLD 4814 Australia; 3 Public Health and Tropical Medicine, College of Public Health, Medical and Veterinary Sciences, James Cook University, 1 James Cook Drive, Douglas, QLD 4814 Australia

**Keywords:** Infectious diseases, Bacterial infection, Influenza virus, Cardiovascular diseases, Heart failure, Vascular diseases

## Abstract

Acute respiratory infections appear to precipitate vascular events. Acute myocardial infarction (AMI) and stroke are the leading cause of death and disability globally. This study was based on a cohort of patients admitted to Townsville University Hospital between January 2006 and December 2016. Using a self-controlled case series design, we investigated the risk of AMI or ischaemic stroke after an episode of pneumonia. We defined the ‘risk interval’ as the first 14 days after hospitalisation for pneumonia and the ‘control interval’ as one year before and one year after the risk interval. Among a population (N = 4557) with a median age of over 70, a total of 128 AMI and 27 stroke cases were identified within 1 year of an episode of pneumonia in this study. Ten and two admissions occurred during the risk interval, while 118 and 25 admissions occurred during the control period. The relative incidence ratios (RIR) of AMI increased after an episode of pneumonia (RIR=4.85, 95% confidence interval (CI) 2.44–9.67). The risk for stroke after the exposure period of 14 days was 4.94 (95% CI 1.12–21.78) considering only the first stroke incidence. The RIR results for AMI and stroke were not altered by adjusting for age, sex or Indigenous status. The risk of AMI and stroke were significantly higher two weeks after an episode of pneumonia.

## Introduction

Cardiovascular disease (CVD) is the number one cause of death globally with an estimated 17.9 million deaths (about one-third of global deaths) in 2016^1^. When combined, the acute vascular events of acute myocardial infarction (AMI) and stroke are the leading cause of death (85% of all CVD deaths) and disability globally^1–5^. The main risk factors for vascular disease are chronic and predominantly associated with lifestyle (smoking and obesity), diabetes, hypertension and familial factors^6–8^. These severe vascular events also appear to be precipitated acutely by lower respiratory tract infections^9,10^. As lower respiratory tract infections (LRTIs) are common, an awareness of the potential for subsequent AMI and stroke is required to direct additional preventive strategies such as vaccination (to prevent pneumonia and viral LRTI) and the use of anti-platelet therapy. The traditional focus on primary cardiovascular risk factor prevention could be complemented by secondary prevention targeted at the post-infective period.

In this study, we sought to quantify the risk of AMI and stroke in patients living in the tropical regions of Northern Australia after an episode of hospitalisation due to lower respiratory tract infections (LRTI), caused by both bacterial pneumonia and influenza. The database used includes a large proportion of Australian Indigenous peoples, so we’re able to explore whether additional factors, including Indigenous status influence this association.

We have employed a self–controlled case series methodology to optimally investigate associations between transient exposures and linked events^11,12^. This methodology allows for minimisation of fixed confounders.

## Results

**Figure 1:**
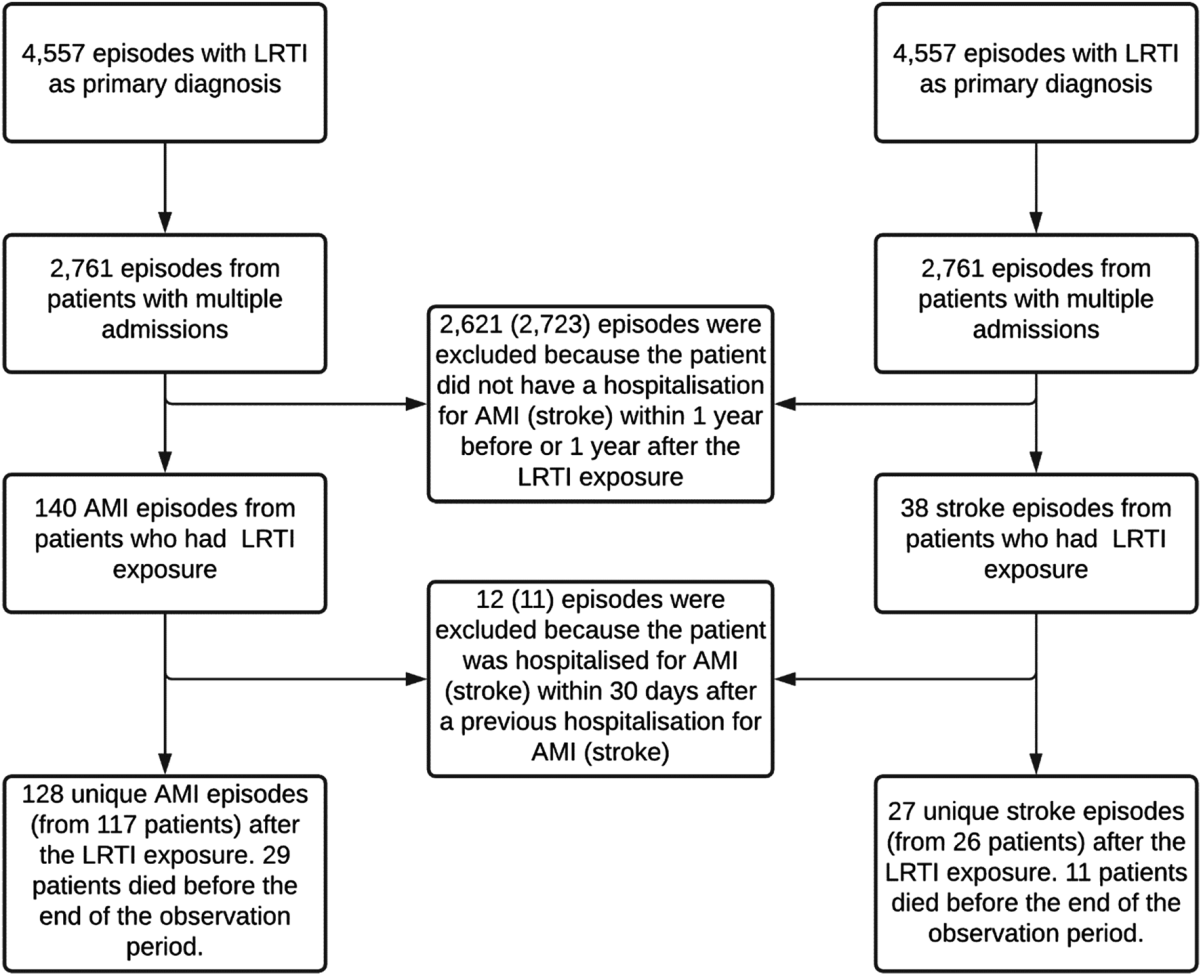
AMI and Stroke admissions included in the study.

Out of a total of 4557 admissions with pneumonia as the primary diagnosis, we identified 128 unique AMI admissions from 117 patients and 27 unique stroke admissions from 26 patients, which occurred within an individual 2-year period determined by the discharge date of pneumonia-related hospitalisation (Fig. 1). Table 1 presents the baseline characteristics of the included patients who had an AMI/stroke episode associated with severe LRTI exposure and those with LRTI only during the 2-year period requiring hospitalisation. Our study population consists of predominantly older adults with a mean age of 77.2 years, 69.4 years and 68.3 years, respectively, for LRTI-AMI, LRTI-stroke and LRTI cohorts. Females accounted only for 42.7% of patients who had AMI after LRTI exposure, but there were more females than males with stroke after LRTI exposure (57.7%) and 47.6% among LRTI only patients. A significant minority (n = 38, 32.5%) of patients identified themselves as Aboriginal and Torres Strait Islander (Indigenous) peoples in the LRTI-AMI and a lesser number in the LRTI-stroke (n = 3, 11.5%) cohorts. The majority of patients were admitted through the hospital’s emergency department (ED), 87 (74.4%) for AMI, 24 (92.3%) for stroke and 549 (71.0%) for LRTI only. When considering conventional risk factors for cardiovascular disease, many of the patients in the LRTI-AMI and LRTI-stroke smoked (74.4% vs 50.0%), (69.2% vs 50.0%) were hypertensive and (7.0% vs 0%) had dyslipidaemia, while 54.7% and 38.5% had diabetes, respectively. These statistics were higher for patients with LRTI only, 70.8% smoked, 70.2 were hypertensive, and 18.1 had dyslipidaemia. Aside from a difference in patient’s age (older) and length of stay (longer), those who had AMI/stroke associated with LRTI exposure during the 2-year period were otherwise not different from patients who did not suffer these diseases.

**Table 1:** Baseline characteristics of patients who had an AMI/stroke during 2-year period associated with LRTI exposure at the first event.

Baseline characteristic	LRTI–AMI	LRTI–stroke	LRTI only
Number of patients, n	117	26	773
Events within 14 days interval	9	2	NA
LOS in days: mean ± SD	7.2 ± 7.3	14.0 ± 15.0	5.7 ± 9.0
Age in years: mean ± SD	69.4 ± 15.4	77.2 ± 10.7	68.3 ± 16.2
**Age group, n (%)**			
20–39 years	1 (0.9)	0 (0.0)	48 (6.2)
40–59 years	34 (29.1)	2 (7.7)	161 (20.8)
60–84 years	57 (48.7)	18 (69.2)	442 (57.2)
85+ years	25 (21.4)	6 (23.1)	122 (15.8)
**Gender, n (%)**			
Male	67 (57.3)	11 (42.3)	405 (52.4)
Female	50 (42.7)	15 (57.7)	368 (47.6)
**Indigenous status, n (%)**			
Indigenous	38 (32.5)	3 (11.5)	140 (18.1)
Non–indigenous	79 (67.5)	23 (88.5)	633 (81.9)
**Comorbidity, n (%)**^**a**^			
Hypertension	81 (69.2)	13 (50.0)	543 (70.2)
Dyslipidaemia	7 (6.0)	0 (0)	140 (18.1))
Diabetes	64 (54.7)	10 (38.5)	337 (43.6)
Smoking	87 (74.4)	13 (50.0)	547 ( 70.8)
**Admission source, n (%)**			
Emergency department	87 (74.4)	24 (92.3)	549 (71.0)
Outpatient department	2 (1.7)	0 (0.0)	113 (14.6)
Transferred from another hospital	17 (14.5)	1 (3.8)	21 (2.7)
Others	11 (9.4)	1 (3.8)	90 (11.6)

*LOS* Length of hospital stay.

^a^Percentages of presence of comorbidity vs. absence of comorbidity.

**Figure 2:**
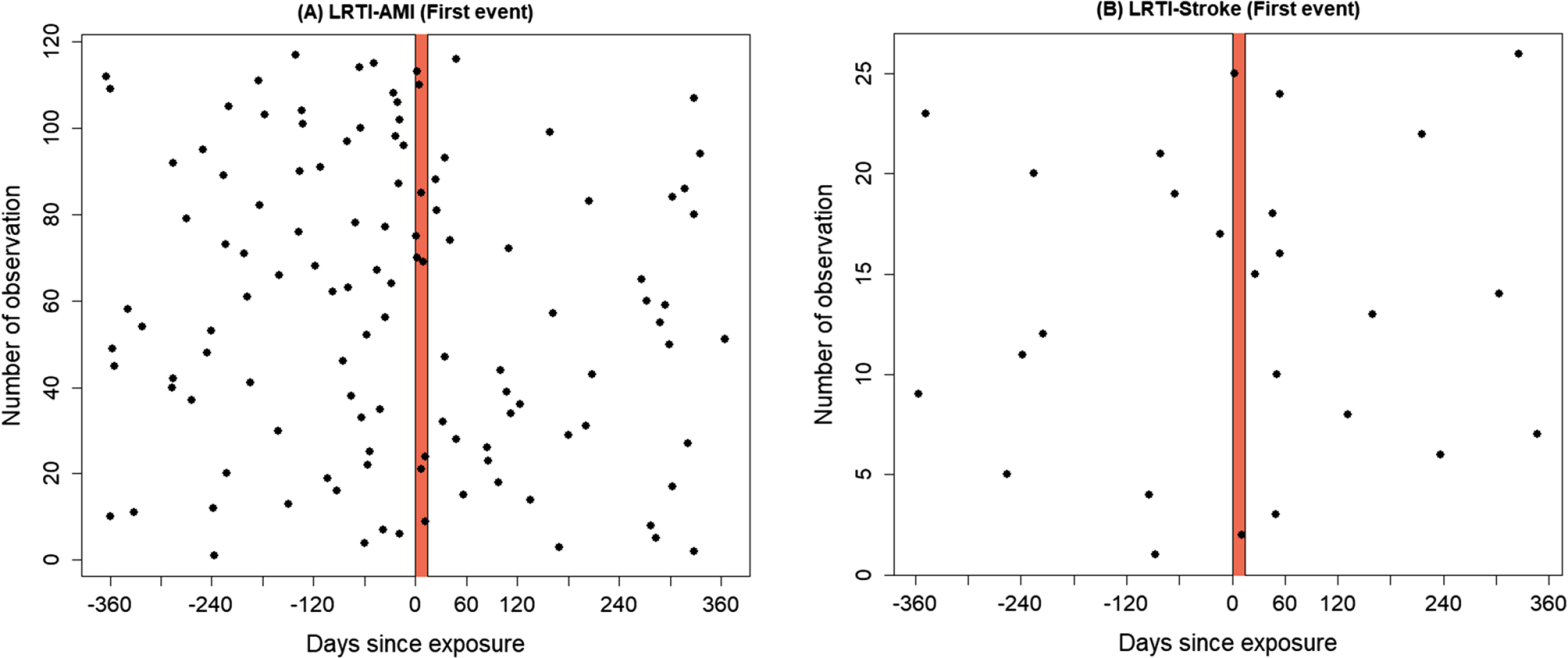
Plot of individual patient-level observation period and the 14 days LRTI risk periods (shaded). Days since exposure is the number of days before or after hospitalisation for LRTI.

We found strong evidence of an increased risk of AMI after hospitalisation for LRTI, with the most substantial effect in the first 2 weeks. We recorded nine cases of AMI occurring in the “at-risk” period after exposure and 108 episodes in the control period (Fig. 2A). The results in Table 2 showed that the RIRs for AMI in Model I were 4.85 (95% CI 2.44–9.67) and 4.91 (95% CI 2.55–9.46) for first AMI events and all AMI events respectively.

**Table 2:** Relative incidence ratios (RIR) for AMI/stroke after LRTI exposure with sensitivity/robustness checks.

Model	First AMI events only n = 117	All AMI events n = 128	First stroke events only n = 26	All stroke events n = 27
**Model 1**				
Exposure 1–14 days	4.85*** (2.44–9.67)	4.91*** (2.55–9.46)	4.94** (1.12–21.78)	4.61** (1.05–20.22)
**Model 2 (with extended exposure period)**				
Exposure 1–14 days	4.85*** (2.43–9.67)	4.90*** (2.54–9.44)	5.12** (1.16–22.70)	4.76** (1.08–20.98)
Exposure 15–28 days	0.98 (0.24–3.97)	0.88 (0.22–3.57)	2.21 (0.29–16.95)	2.10 (0.28–15.97)
**Model 3**				
Exposure 1–91 days	1.63* (1.00–2.66)	1.51* (0.94–2.44)	3.94*** (1.62–9.56)	3.54*** (1.48–8.46)
**Sensitivity checks for Model 1**				
Control for pre-exposure period (7 days)	4.79*** (2.41–9.55)	4.85*** (2.52–9.35)	4.87** (1.10–21.46)	4.54** (1.04–19.93)
Control for pre-exposure period (14 days)	4.74*** (2.38–9.44)	4.85*** (2.52–9.34)	4.80** (1.09–21.18)	4.48** (1.02–19.65)
Exclude who died within 90 days of AMI/stroke	4.70*** (2.27–9.75)	4.25*** (2.06–8.80)	3.32 (0.42–26.18)	3.01 (0.39–23.46)

Robustness check for Model 1	First cancer events only N = 106	All cancer events only N = 146	First diabetes events only N = 110	All diabetes events N = 178
Exposure 1–14 days	1.77 (0.65–4.85)	1.28 (0.47–3.46)	1.47 (0.46–4.63)	1.21 (0.45–3.26)

***Significant at 1%; **significant at 5%; *significant at 10%.

Considering the first event only, there were two admissions for stroke during the “at-risk” 14–day interval after the exposure and 24 stroke episodes during the control period (Fig. 2B). Although the number of patients with a stroke episode was small which widened the confidence intervals of the estimates, we found that the rates of stroke were substantially higher after the exposure to LRTI as presented in Table 2. The relative incidence ratios (RIR) for the main models (Model 1) with the exposure period of 14 days were 4.94 (95% CI 1.12–21.78) and 4.61 (95% CI 1.05–20.22) accounting for first stroke events and all stroke events respectively. For the models with an additional 14-day exposure period (Model 2), the incidence ratios of 5.12 (95% CI 1.16–22.70) and 4.76 (95% CI 1.08 – 20.98) for the interval of 1–14 days were statistically significant for first stroke events and all stroke events, respectively. However, we observed no statistically significant increase in the relative incidence ratios in the longer exposure interval of 15–28 days. Furthermore, in Model 3, the exposure period was extended to 3 months and RIRs were 1.63 (95% CI 1.00–2.66) and 3.94 (95% CI 1.62–9.56) for the first AMI and stroke events, respectively.

To test the validity of our results, we performed sensitivity and robustness checks. The sensitivity analysis showed that our results were largely robust to a different model and sample specifications (Table 2). Changing the control interval by assigning a 7 and 14-day pre-exposure period slightly reduced the incidence ratios for both LRTI-AMI and LRTI-stroke. However, excluding seven stroke episodes with the date of death within 90 days made the results for LRTI-stroke insignificant, which was likely due to the reduction in sample size. For the model which accounted for patients who died shortly after AMI, the results were robust, although the confidence intervals are slightly wider.

In addition to sensitivity analysis, we performed robustness checks by examining associations between LRTI exposure and other health outcomes such as admissions to hospital for complications of diabetes and cancer diagnosis (Table 2). Similar to Kwong et al.^13^, we identified hospital admissions related to diabetes with complications using primary diagnosis based on ICD-10-AM codes E10, E11, E13, and E14. The number of patients in the sample was similar to the number of AMI patients. The RIRs for LRTI-diabetes first event (1.47, 95% CI 0.46–4.63) and LRTI-diabetes all events (1.21, 95% CI 0.45–3.26) were not statistically significant. Similarly, we found no evidence of an increased risk of cancer (ICD-10-AM codes C00-C75 excluding only C34 that encodes for lung cancer) after LRTI.

We explored the heterogeneity in the incidence ratios due to LRTI exposure with respect to gender, age, and indigenous status by including an interaction term (Table 3). We also performed a likelihood ratio test to test whether or not an interaction term should be included in the model. For stroke patients, we did not observe a significant interaction between exposure period and Indigenous status, and no results were reported for models with heterogeneous effects for age and Indigenous status because no variation was observed in the variables due to the small sample size (Table 3).

**Table 3:** Models with interaction for comparing relative incidence ratios for AMI/stroke after LRTI exposure among subgroups.

Model	All AMI events	First AMI events only	All stroke events	First stroke events only
**Model 1 with gender interaction**				
Exposure 1–14	7.41*** (3.10–17.70)	6.83*** (2.87–16.25)	3.33 (0.42–26.23)	3.41 (0.43–26.88)
Exposure 1–14 × male	0.39 (0.09–1.67)	0.51 (0.13–1.93)	2.21 (0.11–42.81)	2.56 (0.13–50.49)
**Model 1 with age interaction**				
Exposure 1–14	6.74*** (2.64–17.21)	5.86*** (2.31–14.86)	NA	NA
Exposure 1–14 × age ≥ 65 years	0.53 (0.13–2.12)	0.72 (0.20–2.65)	NA	NA
**Model 1 with indigenous interaction**				
Exposure 1–14	3.97*** (1.59–9.91)	4.45*** (1.92–10.32)	NA	NA
Exposure 1–14 × indigenous	1.69 (0.42–6.79)	1.30 (0.34–4.95)	NA	NA

***Significant at 1%.

## Discussion

In this study, we provide evidence that, in a population of elderly patients living in the Australian tropics, there is a transient increase in the risk of AMI or stroke after hospitalisation for severe LRTI. The “at-risk” interval, in which the increased risk of AMI/stroke persisted, was 14 days. The incidence of admissions for AMI and stroke were more than four and seven times, respectively, during the 14–day interval after the LRTI stimulus. Our results also showed that an elevated incidence ratio is still present up to 3-months. However, the magnitude of relative risks was smaller than RIRs for 14-day period, which was in line with our expectations. Our findings are consistent with the previous studies suggesting respiratory infections, including pneumonia and influenza, might act as triggers for AMI and stroke events^14,15^. These studies recorded significant increases in incidence risks of stroke following infections with *Streptococcus pneumonia* and influenza.

We also tested whether incidence ratios are heterogeneous with respect to sociodemographic characteristics, such as gender, age, and indigenous status. Although we reported the differences in the incidence ratio point estimates, the analyses had low power to identify statistically significant differences between the subsamples. This is similar to the previous studies where confidence intervals for the estimated incidence ratios stratified by age overlapped and no evidence of significant heterogeneity were reported^14,15^.

From the clinical perspective, both AMI and stroke occur due to atherosclerotic disease and, particularly, unstable vascular plaques. The altered physiology of sepsis with an increased sympathetic tone, vasoconstriction, increased myocardial oxygen demands and hypovolaemia play major roles in precipitation of acute vascular events, and places increased demand on the heart^16–18^. Additionally, atherosclerotic disease has an inflammatory component that is enhanced by severe infection due to LRTI and other infections including urinary tract infections contribute to increased risk of these vascular events through a number of complex mechanisms^19^. Sepsis induces a procoagulant state through the action of cytokines, disturbance in coagulation pathway factors, activated platelets, and innate immune system activation^20^. Prevention of AMI and stroke that occur hyper-acutely in sepsis due to LRTI relies on restoring the normal physiological state with fluid resuscitation, supplemental oxygen, and antibiotics. Other interventions may be useful in the prevention of vascular events during the 2–4 weeks after LRTI. Long term use of aspirin has been associated with reduced cardiovascular events in cohort studies of patients with pneumonia^21^.

A randomised controlled trial of aspirin for the prevention of acute coronary syndrome (ACS) following pneumonia was reported in 2013^22^. This Turkish trial of 185 patients showed a large reduction in the incidence of ACS in patients randomised to 300 mg aspirin daily in the month after a pneumonic illness. Death due to acute coronary syndrome was also significantly reduced. This single trial requires validation with a larger study. Further analysis of the patients involved in the ANTISEPSIS primary prevention trial may be able to provide more evidence of benefit from low-dose aspirin for the prevention of coronary vascular diseases following pneumonia and other sepsis leading to hospitalisation^23^.

Statin drugs have also been studied and may reduce ACS due to lipid-lowering and their immunomodulating effects^24^. A meta-analysis of observational studies shows that the use of statins reduces all-cause mortality after pneumonia^25^. Influenza vaccination appears to reduce mortality due to ACS^26^. Pneumococcal vaccination has also been associated with reduced risk of acute coronary syndromes and associated mortality^27^.

We are cognisant of a number of limitations of our study. Firstly, we did not include the AMI or strokes that occurred during the admission for pneumonia because we were unable to calculate the time interval between these two events if they occurred in the same hospitalisation episode. This potentially underestimated the magnitude of the increased risk of AMI and stroke. The absence of these data also made it difficult to study the periods of risk less than 1 week. Overall, a total of 112 cases of AMI and seven cases of stroke were recorded in discharge ICD-10-AM coding as other diagnoses during a hospital episode where pneumonia was the primary diagnosis.

Secondly, information on subsequent hospitalisations would not be available for patients who may have moved to the catchment of another health services after having an LRTI event. This may lead to the underestimation of our relative risk ratios. However, with the large catchment and no other tertiary hospital within 1300 km, we believe the underestimate will be relatively small compared with other hospitals.

Lastly, the numbers of stroke events included in the study were small and, although significant, the association with preceding LRTI should be interpreted with care as the confidence intervals around the point estimates are wide. While the data used were from a single hospital, potentially limiting generalisation to the Australian population, other similar results from international studies support this result being a true association.

This self-controlled cohort study of patients admitted to an Australian tertiary hospital demonstrates an association between hospitalisation with severe lower respiratory tract infection and increased risk of cardiovascular disease in the 2 weeks post-discharge. There are data from observational cohort studies and randomised controlled trials of benefits of aspirin, statins, influenza and pneumococcal vaccination in the prevention of cardiovascular disease associated with pneumonia. On this basis, it is imperative that optimal secondary prevention strategies are deployed in patients with severe pneumonia and influenza.

## Methods

### Data sources and study design

The data used in this study is derived from a large relational database described in Eisen et al.^28^. The database includes detailed information on the cohort of patients admitted to Townsville University Hospital (TUH) with LRTI between January 1, 2006, and December 31, 2016. TUH is a regional tertiary hospital in Australia providing care for 670,000 people in Northern Australia. The complete database includes diagnostic codes (ICD-10-AM) for 41,367 patients with 378,487 admissions.

### Exposure

We included in our study all inpatients with a primary diagnosis of viral and bacterial pneumonia defined by J09–J18 ICD-10-AM discharge codes. We limit our exposure dates to the period between January 1, 2007, and December 31, 2015, to allow each patient to have a 2-year interval (from 1 year before and 1 year after the exposure date) in which we observe AMI and stroke events.

### Outcome

The outcome of interest in this study was all incidence of AMI (based on ICD-10-AM codes) codes: I21-I22 and ischaemic stroke (I63, G46) between January 1, 2006 and December 31, 2016 occurring in hospitalised patients. The risk event consisted of bacterial pneumonia or influenza occurring as the primary diagnosis for patient hospital admissions. For patients with more than one incident of AMI/stroke within the exposure window, we created two scenarios. The first scenario includes only single incidents of AMI or stroke (the first event for each patient). The second scenario includes the count of all events a patient experienced during the observation period.

**Figure 3:**
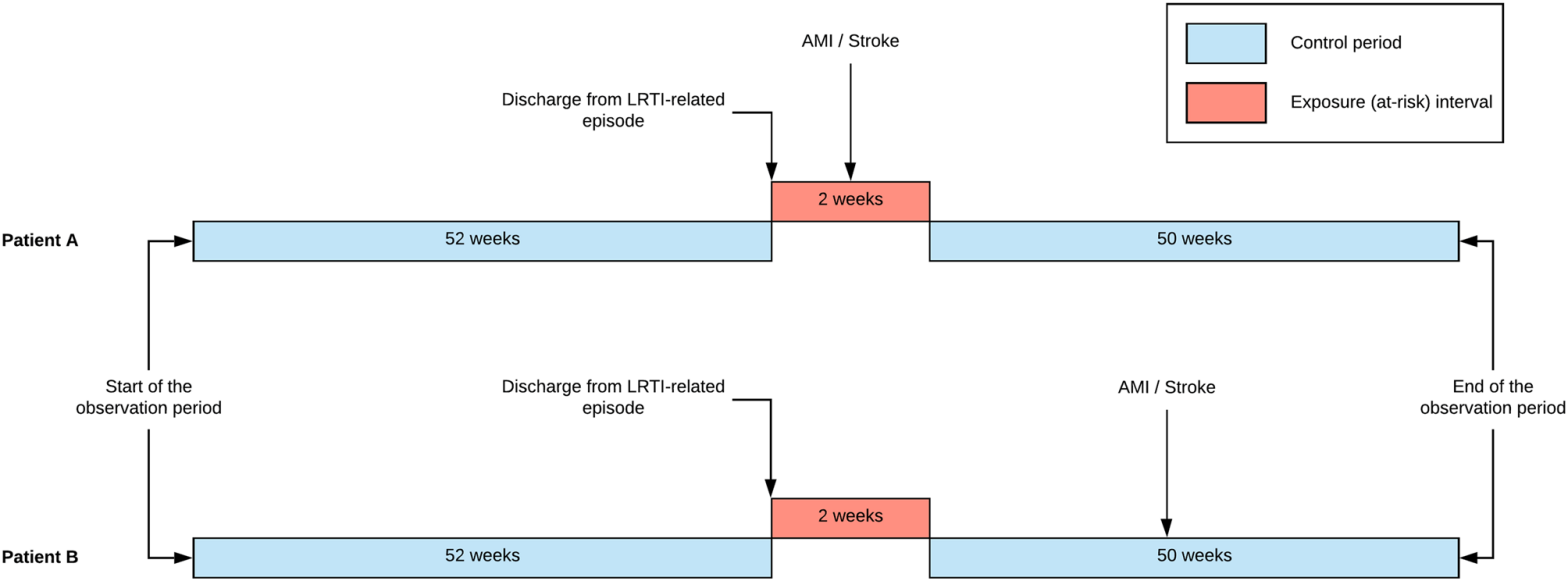
Diagram of the study design.

We define the observation period for each patient as the interval 1 year before and 1 year after the discharge date of the first LRTI exposure. To be included in the study, patients required at least one recorded inpatient AMI/stroke episode within this 2-year period. In the case where an AMI/stroke admission happened within 30 days from the discharge date of the previous episode, we consider the subsequent episode as a related admission and do not count it as a separate event. The schematic of the study design is presented in Fig. 3.

We note that few patients died within the 50-week period after the end of the exposure period. For these patients, the baseline period became smaller, and the observation period ended at the corresponding time of death. Because our study outcomes (AMI and stroke) carry a high mortality risk, the impact of death on the observation periods can create bias in the results, although it is often reported to be small^29^. We test the robustness of our results by running a likelihood ratio test for the models with and without interaction between exposure and censoring indicators. We conclude that this censoring approach does not produce any statistically significant differences and do not cause bias to our results.

### Modelling strategy

We used separate conditional fixed-effects Poisson regressions to compute parameter estimates and interpret the results in terms of incidence rate ratios, i.e. relative incidence of AMI/stroke in “at-risk” or exposure periods to the incidence in baseline or unexposed periods. Because this methodology relies on within-person comparisons, it eliminates the problem of time-invariant (i.e. fixed) confounders. This is advantageous as some confounding factors may not be adequately recorded or captured in the dataset. Furthermore, we account for the time-varying confounders by including controls for calendar months.

Although patient to patient variability (and potential confounding) is avoided by including all individuals in both at risk and control groups, yet some individual-based factors could still modify the risk of the outcome given the exposure.We perform additional analyses by including interaction terms to examine whether heterogeneous effects due to age at AMI/stroke event (< 65 years vs ≥ 65 years), gender (male vs female), and indigenous status (Indigenous vs non-Indigenous) are significant. The likelihood ratio test was used to test the importance of interaction terms.

Experiencing an AMI/stroke may change the probability of subsequent AMI/stroke episodes that might violate the independence of events assumption for the self-controlled case series methodology. To account for this potential problem, we perform a robustness check by running separate analyses for first records of AMI/stroke and all AMI/stroke episodes. We also sought to study the specificity of the demonstrated relationship between the at-risk LRTI disease and AMI/stroke outcome. In this regard, we analysed whether a similar association was present between LRTI and diabetes/cancer, both common non-infective causes for hospitalisation, using the same self-controlled cohort study methodology.

### Ethics approval

This study was part of the project, HREC/16/QTHS/221, approved by the Townsville Hospital and Health Service (THHS) Human Research Ethics Committee. A waiver of consent for access to anonymised data was approved under the Queensland Public Health Act (RD007802). The anonymised secondary data extraction does not require patient recruitment. All methods regarding human use in the manuscript were carried out in accordance with relevant guidelines and regulations.
